# Supplementary material for: Transposable elements-mediated recruitment of KDM1A epigenetically silences HNF4A expression to promote hepatocellular carcinoma
Source: Nat Commun. 2024 Jul 4;15:5631. doi: 10.1038/s41467-024-49926-2 (PMC11224304; doi:10.1038/s41467-024-49926-2)
Supplement: Supplementary file 1 — Supplementary Information [file 41467_2024_49926_MOESM1_ESM.pdf]

Supplementary Figures

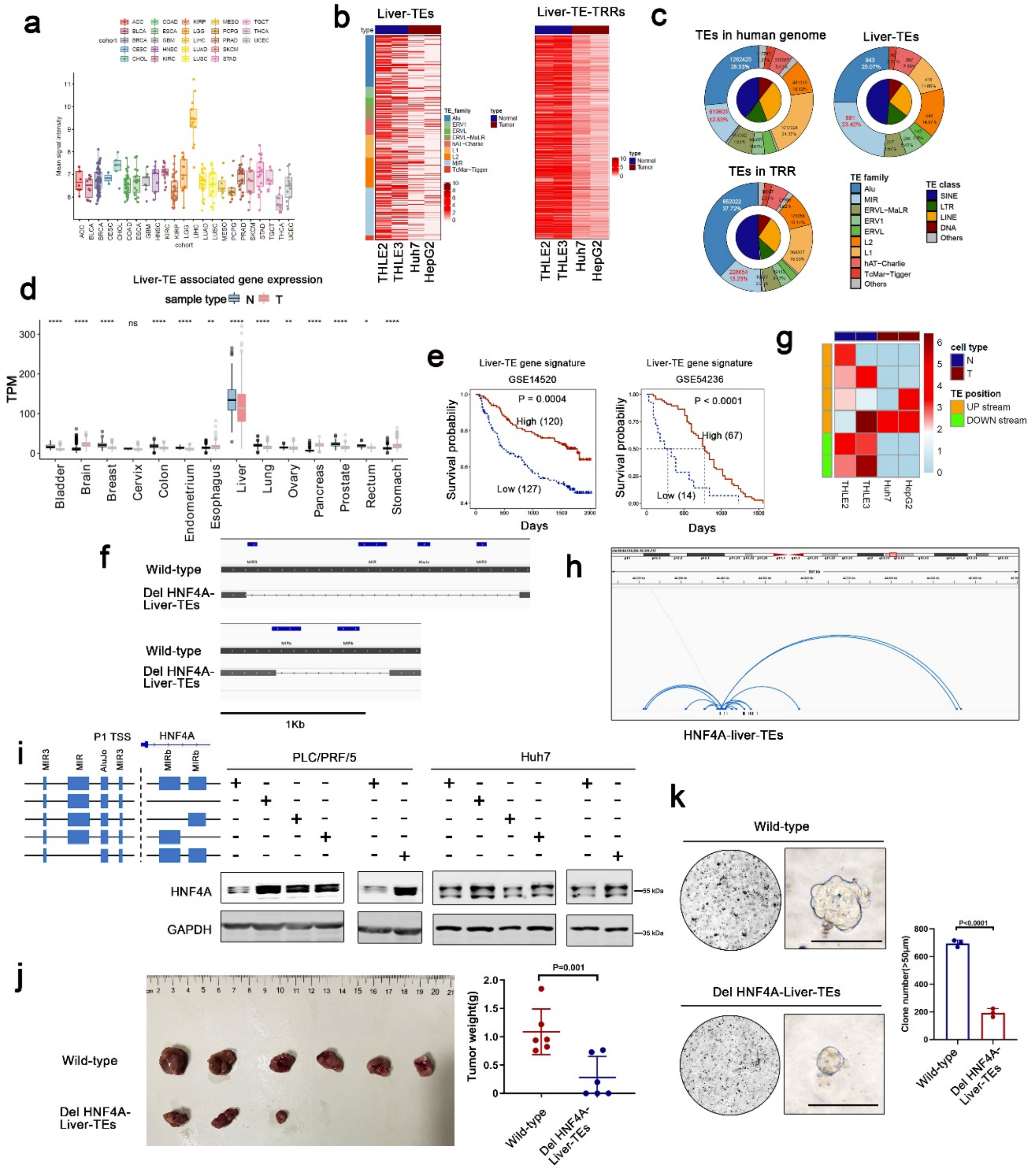

Supplementary figure 1. Correlation of liver-TE-associated genes with favorable prognosis.

(a) Boxplot depicts the mean ATAC-seq signal intensity of liver-TE-TRRs in samples across different tumor types. The centre line represents the median, the bounds of the box indicate the interquartile range, the whiskers extend to the minima and maxima within 1.5 times the IQR from the first and third quartiles. Each point represents the mean intensity in one sample. The ATAC-seq data is obtained from the NIH GDC data portal[1]. (b) The heatmap displays the log-transformed ATAC-seq signal intensity within liver-TEs and liver-TE-TRRs of the healthy liver cells (THLE2, THEL3) and cancerous liver cells (Huh7, HepG2). (c) Proportions of major TE families in the human genome, gene transcription regulatory regions (TRR), and liver-TEs. Chi-square test shows a significant increase in the proportion of MIR family members in liver-TEs ($P<2.2e-16$). (d) Overall expression of liver-TE related genes in different types of normal tissues/cancer tissues. medium, upper and lower quartiles was shown in boxplot. The centre line represents the median, the bounds of the box indicate the interquartile range, the whiskers extend to the minima and maxima within 1.5 times the IQR from the first and third quartiles. t-test, significance was examined by two-sided t test, *$P<0.05$, **$P<0.01$, ***$P<0.0001$, **** $P<0.0001$. The combined GETx-TCGA dataset used in this analysis was obtained from the Xena database [2]. Bladder: N=28, T=407. Brain: N=1157, T=662. Breast: N=292, T=1092. Cervix: N=3, T=304. Colon: N=349, T=288. Endometrium: N=23, T=180. Esophagus: N=666, T=369. Liver: N=160, T=369. Lung: N=397, T=1011. Ovary: N=88, T=419. Pancreas: N=171, T=178. Prostate: N=152, T=495. Rectum: N=10, T=92. Stomach: N=210, T=414. (e) The expression (GSVA score) of liver-TE-associated genes in GSE14520[3] (n=247 samples, High=120, Low=127) and GSE54236[4] (n=81 samples, High=67, Low=14) liver cancer transcriptome datasets is associated with good prognosis of patients. The survival rates were compared using a two-sided log-rank test. (f) Validation of CRISPR/Cas9-mediated deletion of HNF4A-liver-TEs by Sanger sequencing and genome alignment. The BLAST alignment results are displayed in the

IGV browser. (g) The heatmap displays the log-transformed ATAC-seq signal intensity within HNF4A-liver-TEs of the healthy liver cells (THLE2, THEL3) and cancerous liver cells (Huh7, HepG2). (h) The IGV browser illustrates cis-interactions involving HNF4A-liver-TEs in HepG2 cells. The Capture Hi-C data were obtained from the ArrayExpress database (E-MTAB-7144) [5]. (i) HNF4A expression was determined by Western blot assays after CRISPR/Cas9-mediated depletion of the indicated HNF4A-liver-TE. The experiment was repeated twice with similar results. (j) Xenograft experiments conducted in nude mice validated that the depletion of HNF4A-liver-TEs in Huh7 cells effectively suppresses in vivo tumorigenesis. Significance was examined by two-sided t-test, n=6 biological replicates, mean ± SD was shown. (k) Depletion of HNF4A-liver-TEs in primary HCC cells significantly inhibited the organoid formation ability, n=3 biological replicates. Significance was examined by two-sided t-test, mean ± SD was shown. bar=100μm. Source data are provided as a Source Data file.

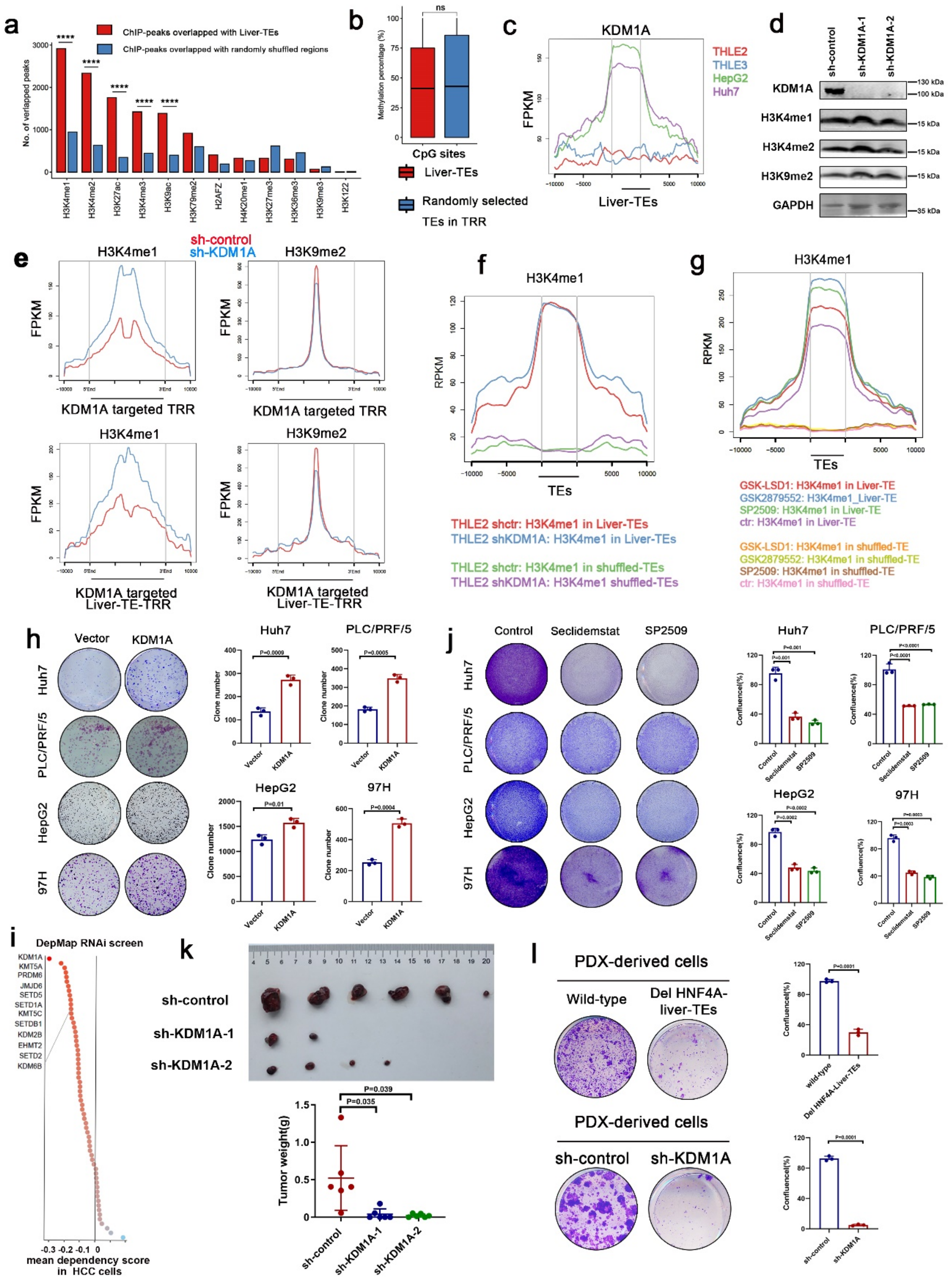
a
ChIP-peaks overlapped with Liver-TEs
ChIP-peaks overlapped with randomly shuffled regions
No. of verlapped peaks
H3K4me1
H3K4me2
H3K27ac
H3K4me3
H3K9ac
H3K79me2
H2AFZ
H4K20me1
H3K27me3
H3K36me3
H3K9me3
H3K122
b
ns
Methylation percentage (%)
CpG sites
Liver-TEs
Randomly selected TEs in TRR
c
KDM1A
THLE2
THLE3
HepG2
Huh7
FPKM
Liver-TEs
d
sh-control
sh-KDM1A-1
sh-KDM1A-2
KDM1A
H3K4me1
H3K4me2
H3K9me2
GAPDH
130 kDa
100 kDa
15 kDa
35 kDa
e
sh-control
sh-KDM1A
H3K4me1
H3K9me2
FPKM
KDM1A targeted TRR
KDM1A targeted Liver-TE-TRR
f
H3K4me1
RPKM
TEs
THLE2 shctr: H3K4me1 in Liver-TEs
THLE2 shKDM1A: H3K4me1 in Liver-TEs
THLE2 shctr: H3K4me1 in shuffled-TEs
THLE2 shKDM1A: H3K4me1 shuffled-TEs
g
H3K4me1
RPKM
TEs
GSK-LSD1: H3K4me1 in Liver-TE
GSK2879552: H3K4me1_Liver-TE
SP2509: H3K4me1 in Liver-TE
ctr: H3K4me1 in Liver-TE
GSK-LSD1: H3K4me1 in shuffled-TE
GSK2879552: H3K4me1 in shuffled-TE
SP2509: H3K4me1 in shuffled-TE
ctr: H3K4me1 in shuffled-TE
h
Vector
KDM1A
Huh7
PLC/PRF/5
HepG2
97H
Clone number
P=0.0009
P=0.0005
P=0.01
P=0.0004
j
Control
Seclidemstat
SP2509
Confluence(%)
i
DepMap RNAi screen
KDM1A
KMT5A
PRDM6
JMJD6
SETD5
SETD1A
KMT5C
SETDB1
KDM2B
EHMT2
SETD2
KDM6B
mean dependency score in HCC cells
k
sh-control
sh-KDM1A-1
sh-KDM1A-2
P=0.039
P=0.035
Tumor weight(g)
l
PDX-derived cells
Wild-type
Del HNF4A-liver-TEs
sh-control
sh-KDM1A
P=0.0001
Confluence(%)

Supplementary figure 2. Epigenetic regulation of liver-TEs by KDM1A.

(a) Analysis of the enrichment level of histone modification markers (HM) on liver-TE elements using ReMapEnrich software, which employed a two-sided binomial test, with p-values adjusted for multiple comparisons using the Benjamini-Yekutieli (BY) method. ****P<0.0001. The exact significance value for each HM was provided in Supplementary Data 3. n=500 iterations in this analysis. (b) The boxplot depicts the percentage of methylated reads in each CpG site for both liver-TEs and a comparably sized sample of randomly selected TEs within TRR regions. The centre line represents the median, the bounds of the box indicate the interquartile range, the whiskers extend to the minima and maxima within 1.5 times the IQR from the first and third quartiles. Statistical significance was determined using the Mann-Whitney U test. n=21542 CpG sites in liver-TEs or randomly selected. The Bisulfite-Seq data was obtained from GEO database under accession GSM1204463 [6] (c) CUT&Tag-seq assays using the KDM1A antibody were performed in healthy liver cell lines (THLE2, THLE3) and HCC cell lines (HepG2, Huh7). n=1 per assay per condition. The Ngsplot depicts the KDM1A binding profiles within liver-TEs and the ±10kb regions surrounding liver-TEs. (d) Western blot assays were performed to assess the effects of KDM1A knockdown on the global levels of H3K4me1, H3K4me2, and H3K9me2 in HepG2 cells. (e) CUT&Tag-seq experiments for H3K4me1 and H3K9me2 were performed in HepG2 cells with KDM1A-knockdown. Ngsplot was utilized to illustrate changes in histone modifications in KDM1A targeted TRR or liver-TE-TRR regions. (f) CUT&Tag-seq assays using the H3K4me1 antibody were performed in THLE2 liver cells. The Ngsplot depicts the H3K4me1 profiles within liver-TEs and the ±10kb regions surrounding liver-TEs. The signal intensity within randomly shuffled TEs were used as control. (g) CUT&Tag-seq assays using the H3K4me1 antibody were performed in HepG2 cells treated with vehicle (control), SP2509 (1μM), GSK-LSD1 (10μM) and GSK2879552 (10μM). The Ngsplot depicts the H3K4me1 profiles within liver-TEs and the ±10kb regions surrounding liver-TEs. (h) Colony formation assays demonstrate the impact of KDM1A overexpression on liver cancer cell growth, n=3 biological replicates. Significance was examined by two-sided t-

test, mean ± SD was shown, ***P<0.001. (i) The mean dependency scores of 62 histone demethylases and methyltransferases in 26 HCC cell lines from DepMap database (RNAi 19Q3)[7]. A negative dependency score means that a gene is essential for cell growth. (j) Colony formation assay was used to evaluate the growth-inhibitory effects of Seclidemstat (1μM) or SP2509 (1μM) on liver cancer cell lines, n=3 biological replicates. Statistical analyses were performed using two-sided t-test, mean ± SD was shown. (k) Xenograft experiments in nude mice confirm that KDM1A knockdown in Huh7 cells inhibits the in vivo tumorigenesis. t-test, n=6 biological replicates, mean ± SD was shown. (l) The HCC-PDX-derived primary cells were subjected to colony formation assays to evaluate the effects of HNF4A-liver-TE-depletion and KDM1A down-regulation, n=3 biological replicates. Statistical analyses were performed using two-sided t-test, mean ± SD was shown. Source data are provided as a Source Data file.

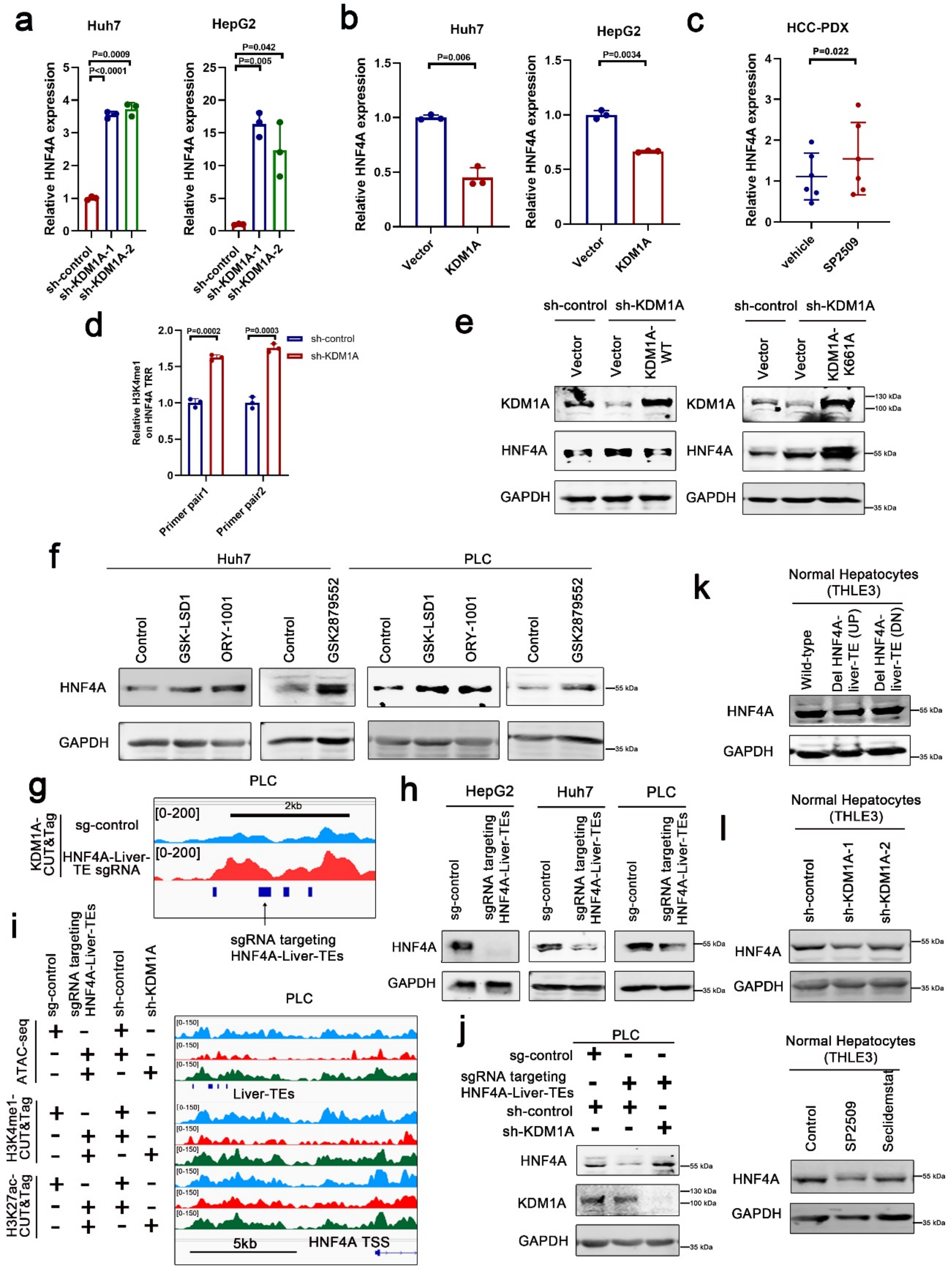
a
Huh7
HepG2
Relative HNF4A expression
sh-control
sh-KDM1A-1
sh-KDM1A-2
P=0.0009
P<0.0001
P=0.042
P=0.005
b
Vector
KDM1A
P=0.006
P=0.0034
c
HCC-PDX
vehicle
SP2509
P=0.022
d
Relative H3K4me1 on HNF4A TRR
Primer pair1
Primer pair2
P=0.0002
P=0.0003
e
KDM1A-WT
KDM1A-K661A
KDM1A
HNF4A
GAPDH
f
PLC
Control
GSK-LSD1
ORY-1001
GSK2879552
g
KDM1A-CUT&Tag
sg-control
HNF4A-Liver-TE sgRNA
[0-200]
2kb
sgRNA targeting HNF4A-Liver-TEs
h
i
ATAC-seq
H3K4me1-CUT&Tag
H3K27ac-CUT&Tag
sh-control
sh-KDM1A
Liver-TEs
5kb
HNF4A TSS
j
k
Normal Hepatocytes (THLE3)
Wild-type
Del HNF4A-liver-TE (UP)
Del HNF4A-liver-TE (DN)
l
Seclidemstat

Supplementary figure 3. KDM1A regulates HNF4A expression.

(a) Real-time PCR showed that KDM1A deletion promoted HNF4A gene expression, n=3 biological replicates. Significance was examined by two-sided t-test, mean ± SD was shown. (b) Real-time PCR showed that KDM1A overexpression decreased HNF4A gene expression, n=3 biological replicates. Significance was examined by two-sided t-test, mean ± SD was shown. (c) Real-time PCR assays were performed on HCC-PDX samples with or without SP2509 treatment to detect HNF4A expression, n=6 biological replicates, Significance was examined by two-sided paired t-test, mean ± SD was shown. (d) ChIP-PCR assays were conducted to measure the relative levels of H3K4me1 modification within the HNF4A transcriptional regulatory region (TRR) in HepG2 cells with KDM1A knockdown, n=3 biological replicates. Significance was examined by two-sided t-test, mean ± SD was shown. (e) Expression of either wild-type or K661A mutant KDM1A in the HepG2 cells with KDM1A knockdown followed by analysis of HNF4A expression through western blot assays. The experiments were repeated two times with similar results. (f) Western blot analysis was carried out to evaluate the expression of HNF4A following treatment with ORY-1001 (10μM), GSK-LSD1 (10μM), or GSK2879552 (10μM). The experiments were repeated three times with similar results. (g) CRISPRa experiments targeting HNF4A-liver-TEs were conducted in PLC/PRF/5 cells, followed by CUT&Tag-seq assays to elucidate KDM1A binding profiles around HNF4A-liver-TEs. n=1 per assay per condition. (h) CRISPRa experiments targeting HNF4A-liver-TEs were conducted in liver cancer cells, followed by western blot detection of HNF4A. The experiments were repeated two times with similar results. (i) ATAC-seq and CUT&Tag-seq assays for H3K4me1 and H3K27ac were were conducted on the specified cell group. The signal intensity around HNF4A-liver-TEs were visualized by IGV browser. n=1 per assay per condition. (j) Knockdown of KDM1A in PLC/PRF/5 cells with HNF4A-targeted CRISPRa attenuated its regulatory influence on HNF4A expression. The experiments were repeated two times with similar results. (k) Western blot assays were used to assess HNF4A expression following CRISPR/Cas9-mediated depletion of

HNF4A-liver-TEs in normal liver cells. The experiments were repeated two times with similar results. (l) HNF4A expression was determined by western blot assays following KDM1A knockdown or KDM1A inhibitor treatment. The experiments were repeated two times with similar results. Source data are provided as a Source Data file.

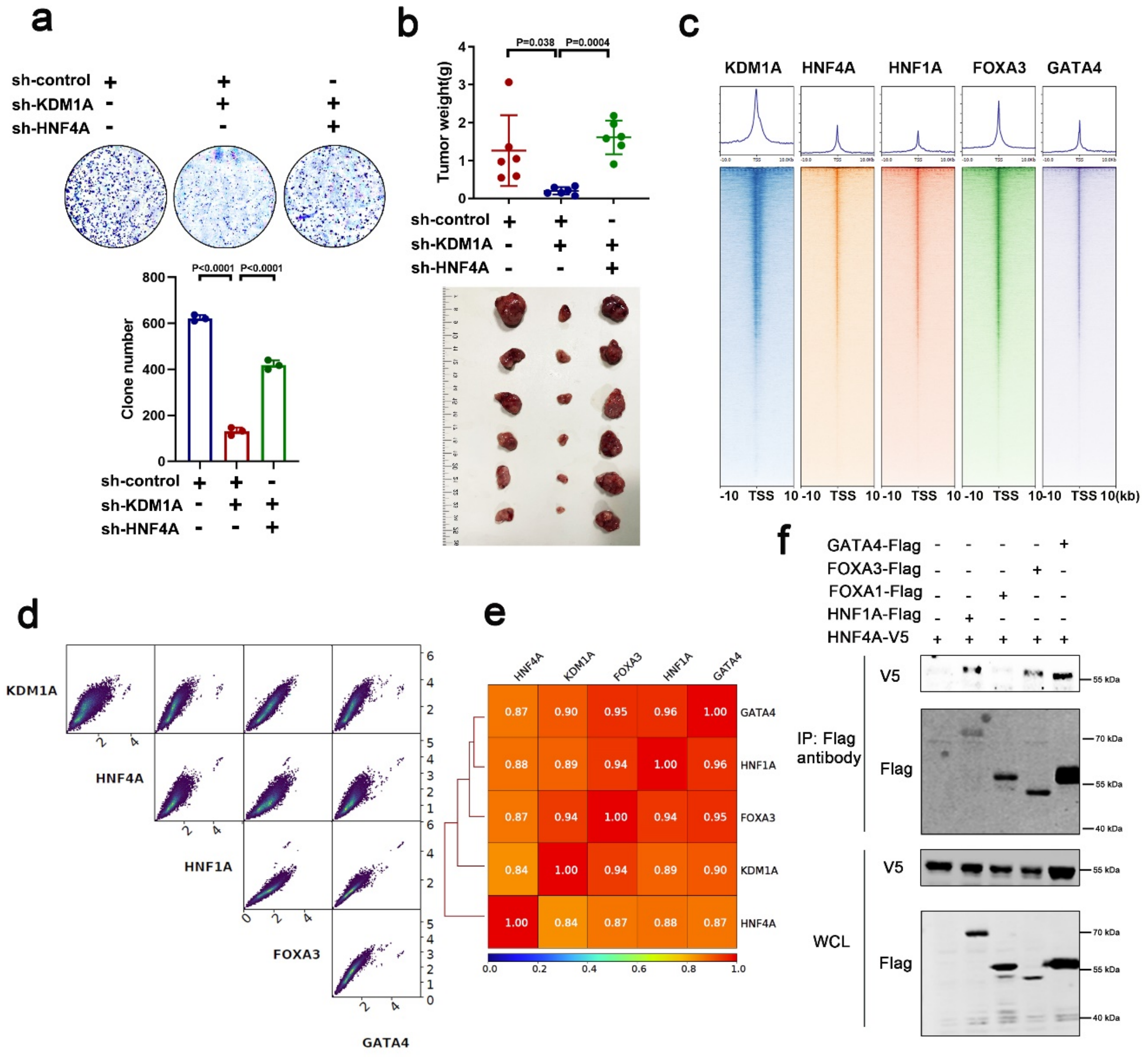

Supplementary figure 4. Interaction of KDM1A with HNF4A and their co-occupancy on the genome.

(a-b) Colony formation (a) and tumorigenesis (b) assays showed that simultaneous knockdown of KDM1A and HNF4A restored both *in vitro* (n=3 biological replicates) and *in vivo* (n=6 biological replicates) cell growth in Huh7 liver cancer cells. Significance was examined by two-sided t-test, mean ± SD was shown. (c-e) Analysis of ChIP-seq

results for KDM1A, HNF4A, HNF1A, FOXA3, and GATA4 in HepG2 cells from the ENCODE database [8, 9]. (c) Heatmap depicting the binding of these proteins at TSS ±10kb regions of genes. (d) Scatter plot showing the Spearman correlation of ChIP-seq signal intensities of these proteins at the gene transcription regulatory regions (TSS ±10kb). (e) Heatmap displaying Spearman correlation coefficients between each pair of proteins in (d). (f) V5-tagged HNF4A protein was expressed in 293T cells along with one of FOXA1-Flag, FOXA3-Flag, GATA4-Flag, and HNF1A-Flag. Co-IP experiments were performed using Flag antibodies, and Western blot was used to detect V5-tagged KDM1A protein that co-precipitated with bait protein. The experiment was repeated twice with similar results. Source data are provided as a Source Data file.

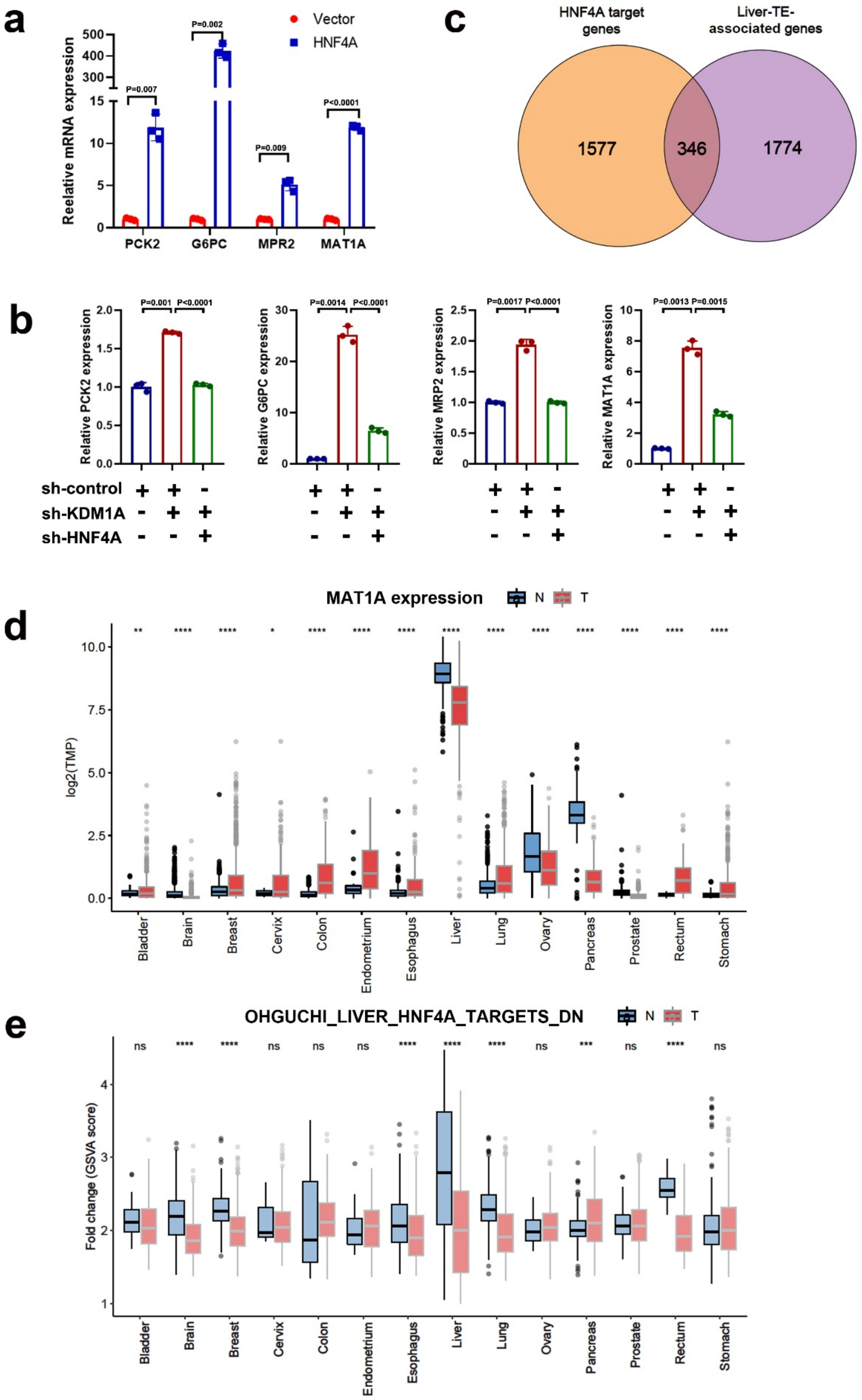
a
Vector
HNF4A
Reelative mRNA expression
P=0.007
P=0.002
P=0.009
P<0.0001
PCK2
G6PC
MPR2
MAT1A
c
HNF4A target genes
Liver-TE-associated genes
1577
346
1774
b
Relative PCK2 expression
Relative G6PC expression
Relative MRP2 expression
Relative MAT1A expression
P=0.001 P<0.0001
P=0.0014 P<0.0001
P=0.0017 P<0.0001
P=0.0013 P=0.0015
sh-control
sh-KDM1A
sh-HNF4A
d
MAT1A expression
N
T
log2(TMP)
Bladder
Brain
Breast
Cervix
Colon
Endometrium
Esophagus
Liver
Lung
Ovary
Pancreas
Prostate
Rectum
Stomach
e
OHGUCHI_LIVER_HNF4A_TARGETS_DN
Fold change (GSVA score)

Supplementary figure 5. Tissue-specific expression of HNF4A down-stream genes.

(a) Real-time PCR experiments were performed to analyze the expression of the indicated genes in Huh7 cells with HNF4A overexpression, n=3 biological replicates. Significance was examined by two-sided t-test, mean ± SD was shown. (b) Real-time PCR experiments were carried out to assess the expression of the indicated genes in Huh7 cells with either KDM1A down-regulation alone or in combination with HNF4A, n=3 biological replicates. Significance was examined by two-sided t-test, mean ± SD was shown. (c) Venn diagram illustrates liver-TE-related HNF4A target genes. (d-e) The expression of *MAT1A* (d) and GSVA score of HNF4A down-stream gene signature (e) was analyzed in various types of normal and cancer tissues, revealing high expression levels in normal liver and liver cancer tissues. However, its expression was found to be significantly down-regulated in liver cancer compared to normal liver tissue, Significance was examined by two-sided t-test, *P<0.05, **P<0.01, ***P<0.0001, **** P<0.0001. In the boxplot, the centre line represents the median, the bounds of the box indicate the interquartile range, the whiskers extend to the minima and maxima within 1.5 times the IQR from the first and third quartiles. The combined GETx-TCGA dataset used in this analysis was obtained from the Xena database[2]. Bladder: N=28, T=407. Brain: N=1157, T=662. Breast: N=292, T=1092. Cervix: N=3, T=304. Colon: N=349, T=288. Endometrium: N=23, T=180. Esophagus: N=666, T=369. Liver: N=160, T=369. Lung: N=397, T=1011. Ovary: N=88, T=419. Pancreas: N=171, T=178. Prostate: N=152, T=495. Rectum: N=10, T=92. Stomach: N=210, T=414. Source data are provided as a Source Data file.

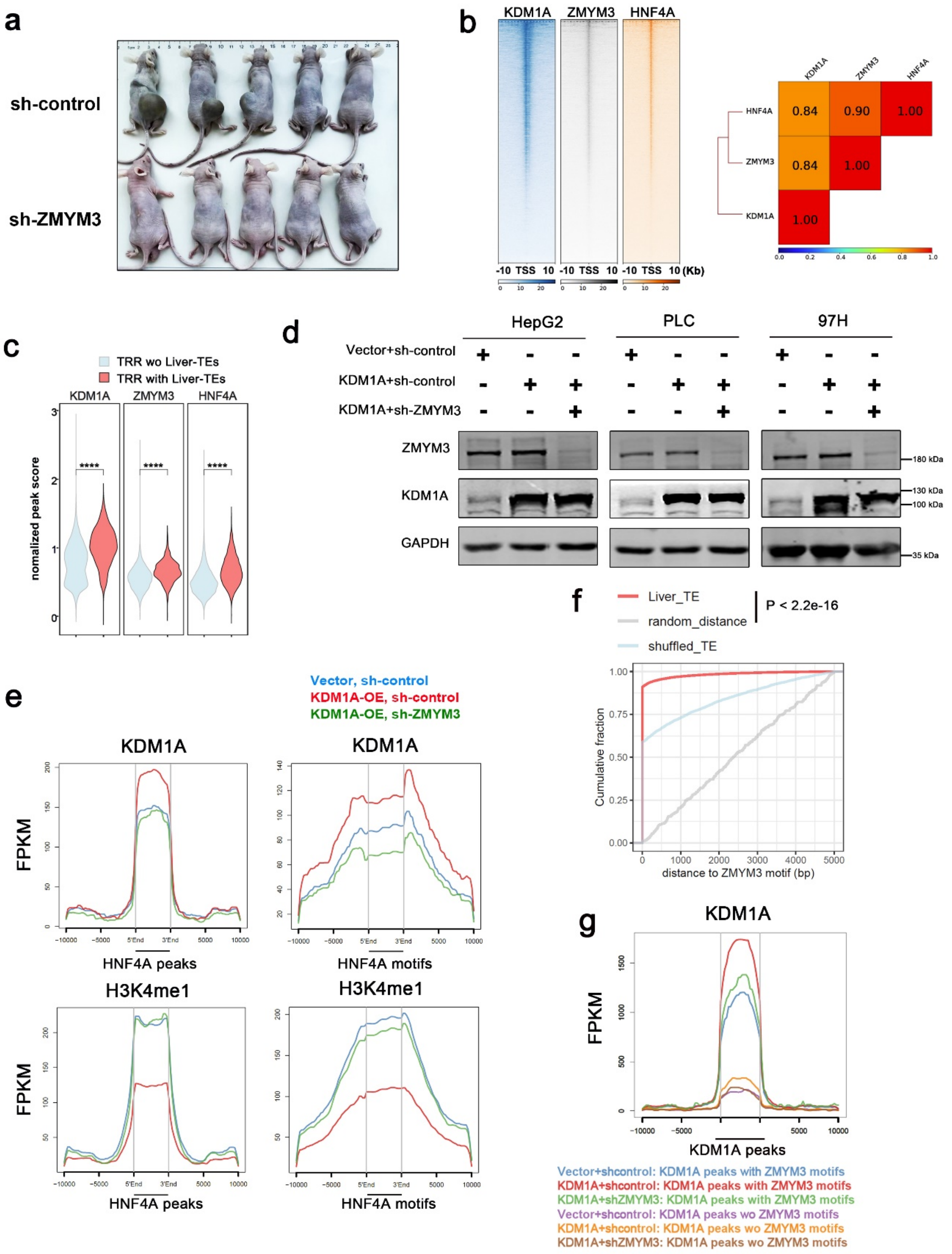
a
sh-control
sh-ZMYM3
b
KDM1A
ZMYM3
HNF4A
-10 TSS 10
(Kb)
0.84
0.90
1.00
c
TRR wo Liver-TEs
TRR with Liver-TEs
nomalized peak score
****
d
HepG2
PLC
97H
Vector+sh-control
KDM1A+sh-control
KDM1A+sh-ZMYM3
ZMYM3
KDM1A
GAPDH
180 kDa
130 kDa
100 kDa
35 kDa
e
Vector, sh-control
KDM1A-OE, sh-control
KDM1A-OE, sh-ZMYM3
FPKM
H3K4me1
HNF4A peaks
HNF4A motifs
f
Liver_TE
random_distance
shuffled_TE
P < 2.2e-16
Cumulative fraction
distance to ZMYM3 motif (bp)
g
KDM1A peaks
Vector+shcontrol: KDM1A peaks with ZMYM3 motifs
KDM1A+shcontrol: KDM1A peaks with ZMYM3 motifs
KDM1A+shZMYM3: KDM1A peaks with ZMYM3 motifs
Vector+shcontrol: KDM1A peaks wo ZMYM3 motifs
KDM1A+shcontrol: KDM1A peaks wo ZMYM3 motifs
KDM1A+shZMYM3: KDM1A peaks wo ZMYM3 motifs

Supplementary figure 6. Co-occupancy of KDM1A, ZMYM3 and HNF4A within transcriptional regulatory regions.

(a) Xenograft experiment in nude mice reveals that ZMYM3 knockdown in Huh7 cells entirely inhibits the *in vivo* tumorigenesis. (b) Analysis of ChIP-seq results for KDM1A, ZMYM3, and HNF4A in HepG2 cells from the ENCODE database [8, 9]. Heatmap depicting the binding of these proteins at TSS ±10kb regions of genes. Spearman correlation of ChIP-seq signal intensities of these proteins at the gene transcription regulatory regions (TSS ±10kb) is shown. n= 45213 genomic regions analyzed using each ChIP-seq data. (c) Comparison of ChIP-seq signal intensities in the TRR regions with and without liver-TEs by t-test, ****P<0.0001. (d) Western blot was used to detect the expression of ZMYM3 and KDM1A across three groups of 97H cells: Vector control + sh-control, KDM1A+sh-control, and KDM1A+sh-ZMYM3. The experiments were repeated twice with similar results. (e) CUT&Tag-seq experiments were performed using KDM1A and H3K4me1 antibodies in three groups of 97H cells: Vector control + sh-control, KDM1A+sh-control, and KDM1A+sh-ZMYM3. Ngsplot was used to display the changes in KDM1A binding strength and H3K4me1 modification within ±10kb of HNF4A binding regions. (f) The cumulative distribution function (CDF) plot depicts the distances between liver-TEs and ZMYM3 motifs. Statistical analysis, using the Wilcoxon test, revealed that the distances between liver-TEs and ZMYM3 motifs were significantly closer compared to those observed between a randomly shuffled TE set and the ZMYM3 motifs ($P < 2.2e-16$). (g) Ngsplot illustrates the KDM1A binding capability in genomic regions with or without ZMYM3 motifs, utilizing CUT&Tag-seq data from Fig. 6e. Source data are provided as a Source Data file.

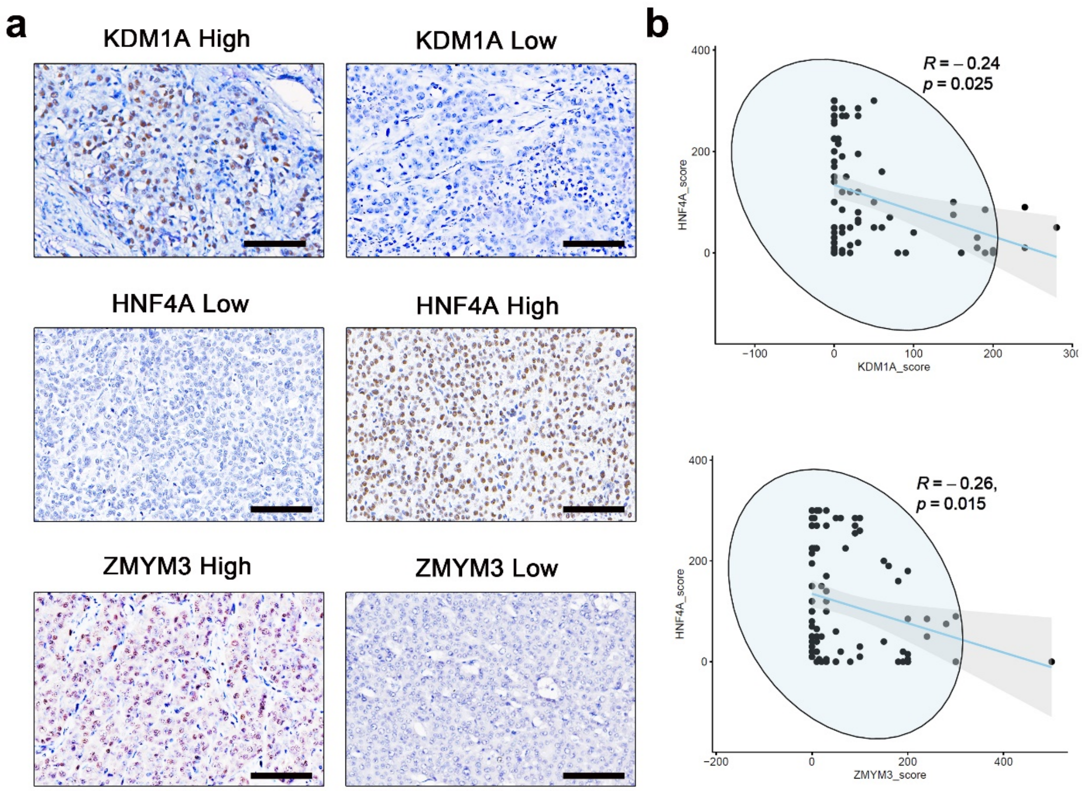

Supplementary figure 7. Related to Figure 7.

(a) Representative immunochemical staining images displaying the expression of KDM1A, HNF4A and ZMYM3 in HCC tissues. bar=100μm. (b) Scatter plots and spearman correlation analyses illustrate negative correlations between the expressions of both KDM1A and ZMYM3 with HNF4A (n=90 independent hepatocellular carcinoma tissue samples). Source data are provided as a Source Data file.

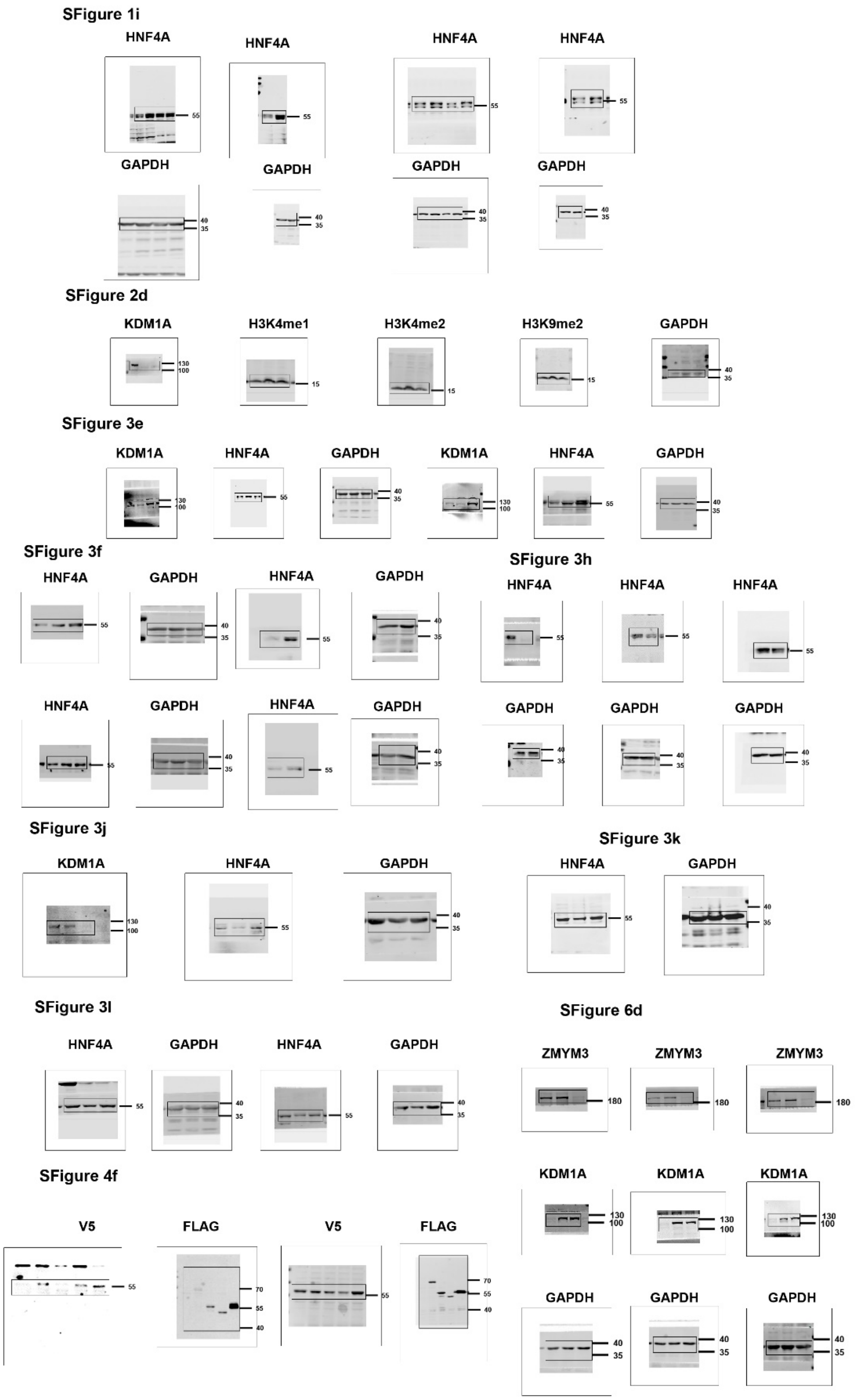

Supplementary figure 8. Original western blots.

Supplementary References

1. Corces MR*, et al.* The chromatin accessibility landscape of primary human cancers. *Science* **362**, (2018).

2. Goldman MJ*, et al.* Visualizing and interpreting cancer genomics data via the Xena platform. *Nat Biotechnol* **38**, 675-678 (2020).

3. Roessler S*, et al.* A unique metastasis gene signature enables prediction of tumor relapse in early-stage hepatocellular carcinoma patients. *Cancer Res* **70**, 10202-10212 (2010).

4. Villa E*, et al.* Neoangiogenesis-related genes are hallmarks of fast-growing hepatocellular carcinomas and worst survival. Results from a prospective study. *Gut* **65**, 861-869 (2016).

5. Chesi A*, et al.* Genome-scale Capture C promoter interactions implicate effector genes at GWAS loci for bone mineral density. *Nat Commun* **10**, 1260 (2019).

6. Ziller MJ*, et al.* Charting a dynamic DNA methylation landscape of the human genome. *Nature* **500**, 477-481 (2013).

7. Tsherniak A*, et al.* Defining a Cancer Dependency Map. *Cell* **170**, 564-576 e516 (2017).

8. Consortium EP. An integrated encyclopedia of DNA elements in the human genome. *Nature* **489**, 57-74 (2012).

9. Partridge EC*, et al.* Occupancy maps of 208 chromatin-associated proteins in one human cell type. *Nature* **583**, 720-728 (2020).
