# Supplementary material for: Transposable elements-mediated recruitment of KDM1A epigenetically silences HNF4A expression to promote hepatocellular carcinoma
Source: Nat Commun. 2024 Jul 4;15:5631. doi: 10.1038/s41467-024-49926-2 (PMC11224304; doi:10.1038/s41467-024-49926-2)
Supplement: Supplementary file 3 — Description of Additional Supplementary Files [file 41467_2024_49926_MOESM3_ESM.pdf]

Description of Additional Supplementary Files

File Name: Supplementary Data 1
Description: The genomic locations of liver-TEs and liver-TE-TRRs.

File Name: Supplementary Data 2
Description: Liver-TE-associated genes.

File Name: Supplementary Data 3
Description: HM enrichment in liver-TEs.

File Name: Supplementary Data 4
Description: TR enrichment in liver-TEs.

File Name: Supplementary Data 5
Description: DepMap analysis results.

File Name: Supplementary Data 6
Description: Differently expressed genes after KDM1A-knockdown.

File Name: Supplementary Data 7
Description: ATAC-seq data accessions from ENOCODE database.

File Name: Supplementary Data 8
Description: Primers used in real-time PCR.
