# Supplementary material for: Transposable elements-mediated recruitment of KDM1A epigenetically silences HNF4A expression to promote hepatocellular carcinoma
Source: Nat Commun. 2024 Jul 4;15:5631. doi: 10.1038/s41467-024-49926-2 (PMC11224304; doi:10.1038/s41467-024-49926-2)
Supplement: Supplementary file 12 — Reporting Summary [file 41467_2024_49926_MOESM12_ESM.pdf]

Corresponding author(s): Boshi Wang, Yanyi Jiang, Yizhen Liu

Last updated by author(s): Jun 22, 2024

Reporting Summary

Nature Portfolio wishes to improve the reproducibility of the work that we publish. This form provides structure for consistency and transparency in reporting. For further information on Nature Portfolio policies, see our Editorial Policies and the Editorial Policy Checklist.

Statistics

For all statistical analyses, confirm that the following items are present in the figure legend, table legend, main text, or Methods section.

n/a	Confirmed	
☐	☒	The exact sample size (*n*) for each experimental group/condition, given as a discrete number and unit of measurement
☐	☒	A statement on whether measurements were taken from distinct samples or whether the same sample was measured repeatedly
☐	☒	The statistical test(s) used AND whether they are one- or two-sided<br>*Only common tests should be described solely by name; describe more complex techniques in the Methods section.*
☒	☐	A description of all covariates tested
☒	☐	A description of any assumptions or corrections, such as tests of normality and adjustment for multiple comparisons
☐	☒	A full description of the statistical parameters including central tendency (e.g. means) or other basic estimates (e.g. regression coefficient) AND variation (e.g. standard deviation) or associated estimates of uncertainty (e.g. confidence intervals)
☐	☒	For null hypothesis testing, the test statistic (e.g. *F*, *t*, *r*) with confidence intervals, effect sizes, degrees of freedom and *P* value noted<br>*Give P values as exact values whenever suitable.*
☒	☐	For Bayesian analysis, information on the choice of priors and Markov chain Monte Carlo settings
☒	☐	For hierarchical and complex designs, identification of the appropriate level for tests and full reporting of outcomes
☐	☒	Estimates of effect sizes (e.g. Cohen's *d*, Pearson's *r*), indicating how they were calculated

Our web collection on statistics for biologists contains articles on many of the points above.

Software and code

Policy information about availability of computer code

Data collection

Identification of liver-TEs
To identify liver-TEs, we obtained RepeatMasker-annotated transposable elements from the UCSC database. We also obtained gene annotation files (gencode.v41) for hg38 genome from the UCSC database to extract transcription start sites (TSS). To assess chromatin accessibility in TRR-TEs across different tissue types, we downloaded ATAC-seq data (bigWig format) from the NIH GDC data portal (https://gdc.cancer.gov/about-data/publications/ATACseq-AWG) . For the ATAC-seq data downloaded from ENCODE database (the accession numbers from ENCODE were listed in Supplementary Data 7).
Publicly available transcriptome and epigenome datasets
The HCC dataset from The Cancer Genome Atlas (TCGA) database (https://portal.gdc.cancer.gov/) was obtained using the TCGAbiolinks package (2.16.4). FPKM-normalized RNA-seq data were converted to TPM values using the TCGA dataset for further analysis. Additionally, the GSE14520 (https://www.ncbi.nlm.nih.gov/geo/query/acc.cgi?acc=GSE14520) and GSE54236 (https://www.ncbi.nlm.nih.gov/geo/query/acc.cgi?acc=GSE54236) HCC transcriptome datasets were obtained from the Gene Expression Omnibus (GEO) database using the GEOquery package (2.56.0). The HCC dataset from the International Cancer Genome Consortium (ICGC) database was obtained from the ICGC data portal (https://dcc.icgc.org/). From the UCSC Xena database (https://xenabrowser.net/), we downloaded a combined TCGA and Genotype-Tissue Expression (GTEx) dataset for analyzing the overall expression of liver-TE associated genes in different types of normal tissues/cancer tissues.
Several publicly available ChIP-seq raw data were downloaded from the ENCODE database (https://www.encodeproject.org/), including ENCFF750VZA (KDM1A-ChIP-seq in HepG2 cells), ENCFF000PJT (HNF4A-ChIP-seq in HepG2 cells), ENCSR848YWD (ZMYM3-ChIP-seq in HepG2 cells), ENCFF442RQA (HNF1A-ChIP-seq in HepG2 cells), ENCFF492CBJ (FOXA3-ChIP-seq in HepG2 cells), and ENCFF163SRP (GATA4-ChIP-seq in HepG2 cells). To analyze the methylation status of CpG sites in liver-TEs, we downloaded a BED file (GSM1204463, https://www.ncbi.nlm.nih.gov/geo/query/acc.cgi?acc=GSM1204463) from the GEO database. This file contained the frequency of methylated reads in each detected CpG site in HepG2 cells, which were obtained using a Reduced Representation Bisulfite Sequencing (RRBS) assay. We shifted

the reference genome from hg19 to hg38 using the liftOver software and used BEDTools (v2.31.1) to extract CpG sites within liver-TEs for comparison of methylation levels with a similarly sized sample of randomly selected TEs within TRR regions. Capture Hi-C data were obtained from the ArrayExpress database (E-MTAB-7144), originating from HepG2 cells.

Data analysis

Identification of liver-TEs

To identify liver-TEs, we followed the following procedure. First, we obtained RepeatMasker-annotated transposable elements from the UCSC database. We also obtained gene annotation files (gencode.v41) for hg38 genome from the UCSC database to extract transcription start sites (TSS). We defined transcriptional regulatory regions (TRR) as genomic regions 10kb upstream and downstream of TSS with a Smith-Waterman score >100. We then restricted TEs for analysis to major TE class/families, including SINE (Alu, MIR), LINE (L1, L2), LTR (ERV1, ERVL, ERVL-MaLR), and DNA (hAT-Charlie, TcMar-Mariner) that overlapped with TRR (TRR-TEs).

To assess chromatin accessibility in TRR-TEs across different tissue types, we downloaded ATAC-seq data (bigWig format) from the NIH GDC data portal (https://gdc.cancer.gov/about-data/publications/ATACseq-AWG) 44 We used the multiBigwigSummary program (parameters: BED-file, --binSize 500) in the deeptools package (3.5.0) to generate the chromatin accessibility landscape, and analyzed RawCounts files using the Rtsne (0.15) to generate a tSNE map. We identified tumor type-specific TE markers using the FindConservedMarkers function, a program embedded in Seurat package (v4.0.2) and limma (3.44.3) package. We used adj.p value < 0.0001 & log2FC > 10 as the cut-off values for the FindConservedMarkers algorithm and adj.p value < 0.0001 & logFC > 2 and B value > 10 for the limma algorithm. We considered TEs filtered by both algorithms as liver-specific accessible TEs (liver-TEs). We defined the TRR regions containing liver-TEs as liver-TE-TRRs. To identify these regions, we utilized the "bedtools intersect" function within the bedtools software (v2.31.1), analyzing bed files containing liver-TEs and TRR regions to get TRRs harboring liver-TEs. Subsequently, the resultant bed files containing liver-TE-TRR regions were processed using the "bedtools merge" function to create a list of non-overlapping liver-TE-TRRs. In this study, we defined liver-TE associated genes as genes that have at least one liver-TE located within 10kb of their transcription start site (TSS). We created a geneset comprising all liver-TE associated genes identified in our analysis. This geneset was subjected to gene set variation analysis (GSVA) and gene set enrichment analysis (GSEA) based on publicly available HCC transcriptome datasets or RNA-seq results generated from our own experiments.

For the ATAC-seq data downloaded from ENCODE database (the accession numbers from ENCODE were listed in Supplementary Data 7). The uniquely mapped reads were isolated by using samtools (v1.6) under parameters: samtools view -f 2 -q 10 -b. Then, the Bam files including uniquely aligned reads were transformed to bigwig formatted files by bamCoverage function in Deeptools (v3.5.1) with default parameters. Then, we used the multiBigwigSummary program (parameters: BED-file, --binSize 500) in the deeptools package (3.5.0) to analyze the chromatin accessibility landscape.

Identification of transcriptional regulators and histone markers enrichment in liver-TEs

To determine the enrichment of transcription regulatory (TR) proteins or histone markers (HM) in liver-TEs, we utilized the Remapenrich package (0.99.0) (https://github.com/remap-cisreg/ReMapEnrich). First, we obtained a bed-formatted file containing the positions of liver-TEs as the query set. For TR enrichment analysis, we downloaded the bed-formatted Remap catalog (2020 version) from the Remap database (https://remap.univ-amu.fr/storage/remap2020/hg38/MACS2 /remap2020_all_macs2_hg38_v1_0.bed.gz)45. We then extracted ChIP-peaks from liver cancer HepG2 cells to assemble a sub-catalog for this analysis. For HM enrichment analysis, we downloaded the HM ChIP-seq peaks of HepG2 cells from the GTRD database (http://gtrd.biouml.org:8888/downloads/20.06/bigBeds/hg38/ChIP-seq_HM/Peaks/) and assembled a custom Remap-catalog formatted file. For both TR and HM enrichment analyses, the software generated background regions by randomly shuffling the query regions within TRR for 500 iterations (shuffles=500). Other parameters in the program were set as follows: byChrom = F, fractionQuery=0.01, fractionCatalog=0.01, included = 0.9, tail="both". The enrichment of TR or HM in liver-TEs was then determined using the Remapenrich package.

To analyze the TRs that enriched in the promoters of a selected set of genes, a Lisa software (https://github.com/liulab-dfci/lisa) was also used. It uses a gene-centric approach to identify transcription factors and other regulatory proteins by integrating online ChIP-seq data.

Publicly available transcriptome and epigenome datasets

The HCC dataset from The Cancer Genome Atlas (TCGA) database (https://portal.gdc.cancer.gov/) was obtained using the TCGAbiolinks package (2.16.4). FPKM-normalized RNA-seq data were converted to TPM values using the TCGA dataset for further analysis. Additionally, the GSE14520 (https://www.ncbi.nlm.nih.gov/geo/query/acc.cgi?acc=GSE14520) and GSE54236 (https://www.ncbi.nlm.nih.gov/geo/query/acc.cgi?acc=GSE54236) HCC transcriptome datasets were obtained from the Gene Expression Omnibus (GEO) database using the GEOquery package (2.56.0). The HCC dataset from the International Cancer Genome Consortium (ICGC) database was obtained from the ICGC data portal (https://dcc.icgc.org/). From the UCSC Xena database (https://xenabrowser.net/), we downloaded a combined TCGA and Genotype-Tissue Expression (GTEx) dataset for analyzing the overall expression of liver-TE associated genes in different types of normal tissues/cancer tissues.

To investigate the prognostic relationship of liver-TE associated genes, we used our created liver-TE gene signature to calculate the GSVA score of liver-TE using the GSVA package. Next, survival analyses were performed on transcriptome datasets containing survival information using the survival (3.2-11) and survminer (0.4.9) packages in R based on the GSVA score in each sample. Cut-off values were estimated using the maxstat package (0.7-25). Furthermore, the maxstat method was also utilized to stratify KDM1A expression for Kaplan-Meir survival analysis.

Several publicly available ChIP-seq raw data were downloaded from the ENCODE database (https://www.encodeproject.org/), including ENCFF750VZA (KDM1A-ChIP-seq in HepG2 cells), ENCFF000PJT (HNF4A-ChIP-seq in HepG2 cells), ENCSR848YWD (ZMYM3-ChIP-seq in HepG2 cells), ENCFF442RQA (HNF1A-ChIP-seq in HepG2 cells), ENCFF492CBJ (FOXA3-ChIP-seq in HepG2 cells), and ENCFF163SRP (GATA4-ChIP-seq in HepG2 cells). To determine protein binding regions based on these data, adapters were trimmed using Trim-galore (0.6.5-1), and reads were aligned to the human genome (UCSC hg38) using BWA (0.7.17). The aligned reads were then processed by MACS2 (v2.2.7.1) for peak calling, and bigwig files for peak visualization in Integrative Genomics Viewer (IGV) were generated using Deeptools (v3.5.1). For the KDM1A-ChIP-seq data, we categorized genes with KDM1A peaks (identified by MACS2, with a score > 200) within a ±10 kb vicinity of their TSS as KDM1A target genes. To analyze the methylation status of CpG sites in liver-TEs, we downloaded a BED file (GSM1204463, https://www.ncbi.nlm.nih.gov/geo/query/acc.cgi?acc=GSM1204463) from the GEO database. This file contained the frequency of methylated reads in each detected CpG site in HepG2 cells, which were obtained using a Reduced Representation Bisulfite Sequencing (RRBS) assay. We shifted the reference genome from hg19 to hg38 using the liftOver software and used BEDTools (v2.31.1) to extract CpG sites within liver-TEs for comparison of methylation levels with a similarly sized sample of randomly selected TEs within TRR regions.

Capture Hi-C data were obtained from the ArrayExpress database (E-MTAB-7144), originating from HepG2 cells. The raw sequencing data underwent preprocessing using the HiC-Pro software (3.1.0). This included alignment to the human hg38 genome, removal of duplicate reads, filtering for valid interactions, and generation of binned interaction matrices. cis-interactions involving liver-TEs were selected using the bedtools (v2.31.1), and visualized by IGV genome browser.

RNA-seq

Total RNA was extracted using TRIzol Reagent (Life Technologies, CA, USA) according to the manufacturer's protocol, and RNA integrity was assessed using an Agilent Bioanalyzer 2100 (Agilent Technologies, CA, USA) to obtain RIN values. RNA with RIN values > 7 was purified using an

RNAClean XP Kit (Beckman Coulter, Inc. CA, USA) and RNase-Free DNase Set (QIAGEN, GmBH, Germany). RNA libraries were prepared for sequencing using a VAHTS Universal V6 RNA-seq Library Prep Kit for Illumina (Vazyme, Nanjing, China), and sequencing was performed on an Illumina HiSeq 2500 system.

Raw RNA-seq data were subjected to quality control using FastQC (v0.11.9) and trimmed using Trim_galore (0.6.5-1). SortMeRNA (4.2.0) was used to remove rRNA reads to generate clean data. The clean reads were aligned to the human reference genome (hg38) using the STAR (2.7.6a) aligner to generate BAM files. Duplicates were removed using Samtools (1.7), and the unique mapped counts were obtained using featureCounts (2.0.1). Differential gene expression analysis was performed using EdgeR (3.30.3), and gene set enrichment analysis (GSEA) and gene function enrichment were performed using the ClusterProfiler package (3.16.1) based on the EdgeR results. The aligned reads (BAM files) were transformed into bigwig files using Deeptools software (v3.5.1) for visualization in Integrative Genomics Viewer (IGV).

CUT&Tag-seq

We used the Hyperactive® Universal CUT&Tag Assay Kit for Illumina (Vazyme #TD903) to prepare DNA library. Briefly, fifty thousand cells were gently resuspended with NE buffer and incubated on ice for 10 min. The nuclei were isolated and conjugated to 10 µL pre-activated concanavalin A-coated magnetic beads. The bead-bound nuclei were then incubated with 50 µL Antibody Buffer containing anti-biotin rabbit mAb (1:100 dilution) overnight at 4°C. Subsequently, the nuclei were resuspended with 100 µL dig-wash buffer containing antibody (1:100 dilution) to bind with the primary antibody or rabbit IgG (control). The nuclei were washed three times with 200 µL of dig-wash buffer and tagmented with pA/G-Tn5 adapter complex. The tagmented DNA was collected using DNA Extract Beads for library preparation. PCR was performed for library amplification for 15 cycles, and the library was purified using VAHTS DNA Clean Beads (Vazyme #N411). The tagmented DNA was sequenced using the Illumina novaseq 6000 platform.

The sequencing reads obtained from the Illumina platform were subjected to quality control using FastQC (v0.11.9) to assess the quality of the reads. Adapter trimming and read filtering were performed using Trim-galore (0.6.5-1) with the following parameters: --phred33 --length 35 --stringency 3. The filtered reads were then aligned to the human genome (UCSC hg38) using Bowtie2 version 2.2.6 with parameters: --local --very-sensitive --no-mixed --no-discordant --phred33. The aligned reads were processed by MACS2 (v2.2.7.1) to call peaks and transformed to bigwig formatted files by bamCoverage function in Deeptools (v3.5.1) with default parameters. To visualize the genomic tracks and peak profiles, the processed data were visualized using the Integrative Genomics Viewer (IGV), Ngsplot (v2.63) or Deeptools plotheatmap function. The differential peaks between groups were determined by the macs2 bdgdiff function with paramter: --cutoff 2.
The primary antibody used in CUT&Tag-seq assays were as followed: anti-KDM1A (abcam, ab129195, 1:100); anti-H3K4me1 (abcam, ab176877, 1:100); anti-H3K4me2 (abcam, ab32356, 1:100); anti-H3K27ac (abcam, ab4729, 1:100) and anti-H3K9me2 (abcam, ab1220, 1:100).

ATAC-seq

The ATAC-seq library preparation was conducted using the Hyperactive ATAC-Seq Library Prep Kit for Illumina (Vazyme, TD711) according to the manufacturer's guidelines. Each sample comprised a total of 5×105 cells. Initially, cells were washed with 500 µl PBS and then centrifuged at 500×g for 5 minutes at room temperature. Subsequently, the cell pellets were resuspended in 50 µl of cold lysis buffer and incubated on ice for 10 minutes to isolate the nuclei. Following centrifugation at 500×g at 4°C for 5 minutes, the nuclei were subjected to a transposition reaction by incubating them with a 50 µl Tn5 transposome/Transposition reaction mix at 37°C for 30 minutes. The tagmentation process was performed within the transposition reaction system. The fragmented/transposed DNA was purified using VAHTS DNA Clean Beads. The purified DNA underwent two washes with 200 µl of fresh 80% ethanol and was finally eluted in 26 µl of Nuclease-free ddH2O. The library amplification protocol followed the program: 72°C for 3 minutes; 95°C for 3 minutes; 12–15 cycles of 98°C for 10 seconds, 60°C for 5 seconds; 72°C for 1 minute; and then held at 12°C. The amplified ATAC-Seq library was further purified using VAHTS DNA Clean Beads. The purified DNA was washed twice with 200 µl of fresh 80% ethanol and eluted in 22 µl of Nuclease-free ddH2O. Finally, all ATAC-seq libraries were sequenced using the Illumina NovaSeq 6000 platform.

The sequencing data obtained from the Illumina platform underwent quality control assessment using FastQC (v0.11.9) to evaluate read quality. Subsequently, adapter trimming and read filtering were performed using Trim-galore (v0.6.5-1) with the following parameters: --phred33 --length 35 --stringency 3. The resulting high-quality reads were aligned to the reference genome (hg38 for human) using Bowtie2 (v2.2.5) with the parameters: --very-sensitive -x 2000. The uniquely mapped reads were isolated by using samtools view function (samtools v1.6) under parameters: -f 2 -q 10 -b.

DepMap database analysis

To investigate the function of histone methylation-related enzymes in hepatocellular carcinoma (HCC) cell viability, we obtained normalized RNAi screening data from the DepMap database (19Q3) via the website: https://depmap.org/portal/download. We selected genes annotated by Gene Ontology (GO) terms GO_HISTONE_DEMETHYLASE_ACTIVITY or GO_HISTONE_LYSINE_N_METHYLTRANSFERASE_ACTIVITY and liver cancer cell lines for analysis. Ultimately, we ranked the mean dependency scores of 62 histone demethylases and methyltransferases in 26 HCC cell lines to evaluate and compare their significance for HCC cell viability. A gene with a negative dependency score is essential for cell growth.

Motif identification

Motifs significantly enriched in the promoter regions of KDM1A-negatively regulated genes were identified using the findMotifs.pl script in the HOMER package (v4.11), with default parameters.

To identify motifs for protein binding or consensus sequences within each family of liver-TEs, BED-formatted files containing genomic regions (MACS2 called peaks, overlapped binding regions or liver-TE regions) were analyzed using the MEME-ChIP program (v5.4.1), with default parameters. During the MEM-ChIP process, the software MEME and STREME were used to identify novel motifs or consensus sequences. The newly identified motifs were then matched to known motifs in the Cis_BP database or a given set of motifs in MEME format using TOMTOM. The FIMO program was used to screen motifs and find their localizations. Finally, all screened GTF files were merged into a single BED file for Ngsplot visualizing of CUT&Tag-seq or ATAC-seq intensity profiles surrounding specific motifs.

For manuscripts utilizing custom algorithms or software that are central to the research but not yet described in published literature, software must be made available to editors and reviewers. We strongly encourage code deposition in a community repository (e.g. GitHub). See the Nature Portfolio guidelines for submitting code & software for further information.

Data

Policy information about availability of data

All manuscripts must include a data availability statement. This statement should provide the following information, where applicable:

- Accession codes, unique identifiers, or web links for publicly available datasets
- A description of any restrictions on data availability
- For clinical datasets or third party data, please ensure that the statement adheres to our policy

Data Availability
The sequencing data generated in this study have been deposited in the Gene Expression Omnibus (GEO) repository under the accession codes:
GSE228075 (RNA-seq) [https://www.ncbi.nlm.nih.gov/geo/query/acc.cgi?acc=GSE228075],
GSE228072 (RNA-seq) [https://www.ncbi.nlm.nih.gov/geo/query/acc.cgi?acc=GSE228072],
GSE255638 (ATAC-seq) [https://www.ncbi.nlm.nih.gov/geo/query/acc.cgi?acc=GSE255638],
GSE255639 (ATAC-seq) [https://www.ncbi.nlm.nih.gov/geo/query/acc.cgi?acc=GSE255639],
GSE228074 (CUT&Tag-seq) [https://www.ncbi.nlm.nih.gov/geo/query/acc.cgi?acc=GSE228074],
GSE228071 (CUT&Tag-seq) [https://www.ncbi.nlm.nih.gov/geo/query/acc.cgi?acc=GSE228071],
GSE228069 (CUT&Tag-seq) [https://www.ncbi.nlm.nih.gov/geo/query/acc.cgi?acc=GSE228069],
GSE228255 (CUT&Tag-seq) [https://www.ncbi.nlm.nih.gov/geo/query/acc.cgi?acc=GSE228255],
GSE255634 (CUT&Tag-seq) [https://www.ncbi.nlm.nih.gov/geo/query/acc.cgi?acc=GSE255634],
GSE255636 (CUT&Tag-seq) [https://www.ncbi.nlm.nih.gov/geo/query/acc.cgi?acc=GSE255636],
GSE255640 (CUT&Tag-seq) [https://www.ncbi.nlm.nih.gov/geo/query/acc.cgi?acc=GSE255640],
GSE255641 (CUT&Tag-seq) [https://www.ncbi.nlm.nih.gov/geo/query/acc.cgi?acc=GSE255641],
and Sequence Read Archive (SRA) under accession codes:
SRP502998 (CUT&Tag-seq) [https://www.ncbi.nlm.nih.gov/sra/?term=SRP502998],
SRP503102 (CUT&Tag-seq and ATAC-seq) [https://www.ncbi.nlm.nih.gov/sra/?term=SRP503102].
The raw data of LC-MS assays are available at integrated proteome resources (iProX) database under the project ID:
PXD043284 [https://proteomecentral.proteomexchange.org/cgi/GetDataset?ID=PXD043284].
The raw data of MS-based metabolomics have been deposited in the National Genomics Data Center under the accession code:
PRJCA026639 [https://ngdc.cncb.ac.cn/bioproject/browse/PRJCA026639].

The published data reused in this study includes GSE14520 (Expression profile by array) [https://www.ncbi.nlm.nih.gov/geo/query/acc.cgi?acc=GSE14520], GSE54236 (Expression profile by array) [https://www.ncbi.nlm.nih.gov/geo/query/acc.cgi?acc=GSE54236], and GSM1204463 (Bisulfite-Seq) [https://www.ncbi.nlm.nih.gov/geo/query/acc.cgi?acc=GSM1204463] from GEO database, and E-MTAB-7144 (Capture Hi-C) [https://www.ebi.ac.uk/biostudies/arrayexpress/studies/E-MTAB-7144] from ArrayExpress database. Several publicly available ChIP-seq raw data were downloaded from the ENCODE database (https://www.encodeproject.org/), including:
ENCFF750VZA (ChIP-seq) [https://www.encodeproject.org/files/ENCFF750VZA/],
ENCFF000PJT (ChIP-seq) [https://www.encodeproject.org/files/ENCFF000PJT/],
ENCSR848YWD (ChIP-seq) [https://www.encodeproject.org/files/ENCSR848YWD/],
ENCFF442RQA (ChIP-seq) [https://www.encodeproject.org/files/ENCFF442RQA/],
ENCFF492CBJ (ChIP-seq) [https://www.encodeproject.org/files/ENCFF492CBJ/],
ENCFF163SRP (ChIP-seq) [https://www.encodeproject.org/files/ENCFF163SRP/]. The ATAC-seq data of non-tumoral tissues were obtained from BioProject PRJNA63443 [https://www.ncbi.nlm.nih.gov/bioproject/PRJNA63443]. The LIHC RNA-seq data from The Cancer Genome Atlas (TCGA) database was derived from the TCGA data portal [https://portal.gdc.cancer.gov/] . We downloaded ATAC-seq data (bigWig format) from the NIH GDC data portal [https://gdc.cancer.gov/about-data/publications/ATACseq-AWG] . The RNA-seq data of HCC samples from the International Cancer Genome Consortium (ICGC) database was obtained from the ICGC data portal [https://dcc.icgc.org/]. From the UCSC Xena database, we downloaded a combined TCGA and Genotype-Tissue Expression (GTEx) dataset [https://xenabrowser.net/datapages/?cohort=TCGA%20TARGET%20GTEx&removeHub=https%3A%2F%2Fxena.treehouse.gi.ucsc.edu%3A443]51.
The remaining data are available within the Article, Supplementary Information or Source Data file.
Source data are provided with this paper.

Research involving human participants, their data, or biological material

Policy information about studies with human participants or human data. See also policy information about sex, gender (identity/presentation), and sexual orientation and race, ethnicity and racism.

Reporting on sex and gender	The sample size of male and female participants is reported. The information was collected from medical records.
Reporting on race, ethnicity, or other socially relevant groupings	The study population consisted of Chinese individuals, and ethnicity was collected through medical records.
Population characteristics	The tissue samples used in this study for IHC assays were sourced from 90 HCC patients, comprising 80 males and 10 females, with ages ranging from 16 to 73 years (median age = 48.5 years).
Recruitment	The samples used in this study were collected from archived samples according to standard protocols at Renji Hospital, Shanghai Jiao Tong University School of Medicine. As this was a retrospective study, no additional recruitment procedures

were conducted.

Ethics oversight | This study was approved by the Ethics Committee of Renji Hospital, Shanghai Jiao Tong University School of Medicine.

Note that full information on the approval of the study protocol must also be provided in the manuscript.

Field-specific reporting

Please select the one below that is the best fit for your research. If you are not sure, read the appropriate sections before making your selection.

☒ Life sciences ☐ Behavioural & social sciences ☐ Ecological, evolutionary & environmental sciences

For a reference copy of the document with all sections, see nature.com/documents/nr-reporting-summary-flat.pdf

Life sciences study design

All studies must disclose on these points even when the disclosure is negative.

Sample size	Sample sizes were chosen based on established practices in the field and previous studies demonstrating that these sizes are sufficient to detect meaningful biological effects. The rationale for choosing these sample sizes is to ensure robust and reproducible results while considering practical constraints such as available resources and ethical considerations. These sample sizes have been deemed adequate based on the consistency and reproducibility of the results obtained.
Data exclusions	No data were excluded from the analyses.
Replication	All experiments, including ChIP-PCR, RT-PCR, western blotting, luciferase reporter assays, immunofluorescence (IF), co-immunoprecipitation (Co-IP), metabolic assays, and in vitro/in vivo functional studies, were independently replicated two or three times with consistent results. The number of replicates performed is detailed in the respective figure legends. Immunohistochemistry (IHC) experiments were conducted on a cohort of 90 HCC tissue samples. Main findings from the high-throughput DNA/RNA sequencing data were validated using other independent sequencing datasets from public repositories.
Randomization	To minimize potential biases, all mice used in this study were randomized into the described experimental groups.
Blinding	For each of the following experiments, data collection and analysis were performed blinded to group allocation to ensure unbiased results: MS assays, CUT&Tag-seq, ATAC-seq, and RNA-seq Experiments: Samples were collected in our lab, and the assay procedures were conducted by a third-party company that did not have access to the sample group information. The analyses were performed by a specialist who did not have access to the exact group information until the analysis was complete. The assays and subsequent data analysis were conducted without knowledge of which samples belonged to which groups.<br>In vitro/ in vivo functional Studies: Cells or animals used in functional assays were conducted by at least two persons. Experimental manipulations and outcome measurements were separately performed without revealing the group identities.<br>IHC Staining: Tissue samples were anonymized. The immunohistochemistry staining and subsequent scoring were performed by researchers blinded to the clinical information and group allocation.

Reporting for specific materials, systems and methods

We require information from authors about some types of materials, experimental systems and methods used in many studies. Here, indicate whether each material, system or method listed is relevant to your study. If you are not sure if a list item applies to your research, read the appropriate section before selecting a response.

Materials & experimental systems

n/a	Involved in the study	
☐	☒	Antibodies
☐	☒	Eukaryotic cell lines
☒	☐	Palaeontology and archaeology
☐	☒	Animals and other organisms
☒	☐	Clinical data
☒	☐	Dual use research of concern
☒	☐	Plants

Methods

n/a	Involved in the study	
☒	☐	ChIP-seq
☒	☐	Flow cytometry
☒	☐	MRI-based neuroimaging

Antibodies

Antibodies used	The following primary antibodies were used for blocking. They are listed as antigen first, followed by supplier and clone/catalog/lot number as applicable.<br>1) anti-KDM1A abcam, Clone EPR6825, Cat ab129195, lot 1018822-16, 1:100 for CUT&Tag, IF and IHC, 1:1000 for Western blots;<br>2) anti-KDM1A ABclonal, Cat A21801, lot 3560844003, 1:200 for IP and 1:1000 for Western blots;<br>3) anti-ZMYM3 Proteintech, Clone AB_2880221, Cat 25742-1-AP, lot C10-027S, 1:100 for IHC, 1:200 for IP and 1:1000 for Western blots;

4) anti-HNF4A R&D, Clone C1 H1415, Cat PP-H1415-00, lot A-2, 1:100 for IHC, 1:200 for IF and 1:1000 for Western blots;
5) anti-HNF4A ABclonal, Clone ARC2794, Cat A20865, lot 3560844003, 1:1000;
6) anti-H3K4me1 abcam, Clone ERP16597, Cat ab176877, lot GR3208750-3, 1:100 for CUT&Tag and 1:5000 for Western blots;
7) anti-H3K4me2 abcam, Clone Y47, Cat ab32356, lot GR253788-33, 1:100 for CUT&Tag and 1:5000 for Western blots;
8) anti-H3K27ac abcam, Cat ab4729, lot 1059037-1, 1:100 for CUT&Tag;
9) anti-H3K9me2 abcam, Cat ab1220[mAbcam 1220], 1:100 for CUT&Tag and 1:5000 for Western blots;
10) anti-FLAG ABclonal, Clone ARC5111-02, Cat AE063, lot 9100026002, 1:200 for IP and 1:5000 for Western blots;
11) anti-MAT1A ABclonal, Clone AB_2764502, Cat A2630, lot 002800101, 1:1000;
12) anti-V5 ABclonal, Clone AMC0506, Cat AE017, lot 9200017002, 1:200 for IP and 1:5000 for Western blots;
13) Flag M2 affinity gel Bimake, Cat B26101;
14) anti-GAPDH Santa Cruz biotechnology, Clone 6C5, Cat sc-32233, lot G3020, 1:5000;
15) Goat anti-rabbit IgG Secondary Antibody LI-COR, Cat 926-3221, lot D31205-5, 1:10000;
16) Goat anti-mouse IgG Secondary Antibody LI-COR, Cat 926-68020, lot D00310-25,1:1000.
17) Goat anti-rabbit IgG (H+L) Cross-Adsorbed Secondary Antibody, Alexa Fluor™ 488 ThermoFisher, Cat A11008, lot 2420731, 1:1000 ;
18) Goat anti-mouse IgG (H+L) Cross-Adsorbed Secondary Antibody, Alexa Fluor™ 594 ThermoFisher, Cat A11020, lot 2306811, 1:1000.

Validation

No customized antibodies were used. Validation data of the antibodies purchased from commercial vendors are available on the manufactures wepsite and datasheets.
1) anti-KDM1A abcam, Clone EPR6825, Cat ab129195, lot 1018822-16, 1:100 for CUT&Tag, IF and IHC, 1:1000 for Western blots.
https://www.abcam.cn/products/primary-antibodies/kdm1lsd1-antibody-epr6825-nuclear-marker-and-chip-grade-ab129195.html
2) anti-KDM1A ABclonal, Cat A21801, lot 3560844003, 1:200 for IP and 1:1000 for Western blots.
https://abclonal.com.cn/catalog/A21801
3) anti-ZMYM3 Proteintech, Clone AB_2880221, Cat 25742-1-AP, lot C10-027S, 1:100 for IHC, 1:200 for IP and 1:1000 for Western blots.
https://www.ptgcn.com/products/ZMYM3-Antibody-25742-1-AP.htm
4) anti-HNF4A R&D, Clone C1 H1415, Cat PP-H1415-00, lot A-2, 1:100 for IHC, 1:200 for IF and 1:1000 for Western blots.
https://www.rndsystems.com/cn/products/human-hnf-4alpha-nr2a1-antibody-h1415_pp-h1415-00
5) anti-HNF4A ABclonal, Clone ARC2794, Cat A20865, lot 3560844003, 1:1000.
https://abclonal.com.cn/catalog/A20865
6) anti-H3K4me1 abcam, Clone ERP16597, Cat ab176877, lot GR3208750-3, 1:100 for CUT&Tag and 1:5000 for Western blots.
https://www.abcam.com/products/primary-antibodies/histone-h3-mono-methyl-k4-antibody-erp16597-chip-grade-ab176877.html
7) anti-H3K4me2 abcam, Clone Y47, Cat ab32356, lot GR253788-33, 1:100 for CUT&Tag and 1:5000 for Western blots.
https://www.abcam.com/products/primary-antibodies/histone-h3-di-methyl-k4-antibody-y47-chip-grade-ab32356.html
8) anti-H3K27ac abcam, Cat ab4729, lot 1059037-1, 1:100 for CUT&Tag.
https://www.abcam.com/products/primary-antibodies/histone-h3-acetyl-k27-antibody-chip-grade-ab4729.html
9) anti-H3K9me2 abcam, Clone mAbcam 1220, Cat ab1220, 1:100 for CUT&Tag and 1:5000 for Western blots. https://www.abcam.com/products/primary-antibodies/histone-h3-di-methyl-k9-antibody-mabcam-1220-chip-grade-ab1220.html
10) anti-FLAG ABclonal, Clone ARC5111-02, Cat AE063, lot 9100026002, 1:200 for IP and 1:5000 for Western blots.
https://abclonal.com.cn/catalog/AE063
11) anti-MAT1A ABclonal, Clone AB_2764502, Cat A2630, lot 002800101, 1:1000.
https://abclonal.com.cn/catalog/A2630
12) anti-V5 ABclonal, Clone AMC0506, Cat AE017, lot 9200017002, 1:200 for IP and 1:5000 for Western blots.
https://abclonal.com.cn/catalog/AE017
13) Flag M2 affinity gel Bimake, Cat B26101.
https://www.selleck.cn/bioreagents/anti-flag-magnetic-beads.html
14) anti-GAPDH Santa Cruz biotechnology, Clone 6C5, Cat sc-32233, lot G3020, 1:5000.
https://www.scbt.com/p/gapdh-antibody-6c5
15) Goat anti-rabbit IgG Secondary Antibody LI-COR, Cat 926-3221, lot D31205-5, 1:10000.
https://www.licor.com/bio/reagents/irdye-800cw-goat-anti-rabbit-igg-secondary-antibody
16) Goat anti-mouse IgG Secondary Antibody LI-COR, Cat 926-68020, lot D00310-25,1:10000.
https://www.licor.com/bio/reagents/irdye-680lt-goat-anti-mouse-igg-secondary-antibody
17) Goat anti-rabbit IgG (H+L) Cross-Adsorbed Secondary Antibody, Alexa Fluor™ 488 ThermoFisher, Cat A11008, lot 2420731, 1:1000.
https://www.thermofisher.cn/cn/zh/antibody/product/Goat-anti-Rabbit-IgG-H-L-Cross-Adsorbed-Secondary-Antibody-Polyclonal/A-11008
18) Goat anti-mouse IgG (H+L) Cross-Adsorbed Secondary Antibody, Alexa Fluor™ 594 ThermoFisher, Cat A11020, lot 2306811, 1:1000.
https://www.thermofisher.cn/cn/zh/antibody/product/Goat-anti-Mouse-IgG-H-L-Cross-Adsorbed-Secondary-Antibody-Polyclonal/A-11020

Eukaryotic cell lines

Policy information about cell lines and Sex and Gender in Research

Cell line source(s)

PLC/PRF/5 (CRL-8024), HepG2 (HB-8065), THLE2 (CRL-2706), THLE3 (CRL-3583) and HEK293T (CRL-3216) cells were acquired from the American Type Culture Collection (ATCC, Manassas, VA, USA). Huh7(SCSP-526) cells were obtained from the Cell Bank of the Chinese Academy of Sciences (Shanghai, China). MHCC97H (97H) cells were provided by the Liver Cancer Institute of Zhongshan Hospital, Fudan University (Shanghai, China).

Authentication	Cell lines were tested by short tandem repeat (STR) profiling which compared to the ATCC STR database to verify authentication.
Mycoplasma contamination	All cells were routinely tested using a mycoplasma detection kit (C0301S, Beytime) to ensure no mycoplasma contamination.
Commonly misidentified lines (See ICLAC register)	None

Animals and other research organisms

Policy information about studies involving animals; ARRIVE guidelines recommended for reporting animal research, and Sex and Gender in Research

Laboratory animals

Animal studies

BALB/c nude mice (6 weeks old) were obtained from SLAC (Shanghai, China) and cultured under pathogen-free conditions in Shanghai Jiao Tong University Laboratory Animal Center. The mice were housed in a controlled environment with a 12-hour light/dark cycle. The ambient temperature was maintained within the range of 20-26°C, while humidity levels were kept between 40% and 70%. Daily welfare monitoring was conducted to assess their health, behavior, and any signs of distress, ensuring their well-being throughout the study period. All animal experiments were conducted in accordance with the guidelines approved by Institutional Animal Care and Shanghai Jiao Tong University Animal Care commission. The maximal tumor size/burden permitted is 2000 mm3. To ensure humane endpoints, mice were euthanised using carbon dioxide (CO_2) asphyxiation followed by cervical dislocation to ensure death.

In vivo tumorigenesis assay

For in vivo tumorigenesis assays, 2 × 106 Huh7 cells expressing either vector control or specified shRNA was subcutaneously injected into male nude mice. At the end of the study, tumors were excised, weighed and photographed.

Patient-derived xenografts (PDX)

The HCC patient-derived xenografts (HCC-PDX, No. LI0024, female) was obtained from Shanghai GeneChem Organism by implanting tumor sample fragments (20-30 mm3) derived from an HCC patient into female nude mice (6 weeks old). After one month, twelve mice with tumors reaching an average size of approximately 163 mm3 were selected and randomly divided into 2 groups: six mice in the control group received intraperitoneal injection of 10µl/g vehicle (10% DMSO, 90% corn oil), and six mice in the test group received SP2509 treatment (i.p., 10µl/g, 25 mg/kg, twice a week for 3 weeks). Subsequently, tumor xenograft volumes and mice body weights were measured every three days for three weeks. The formula for calculating tumor volume was V = 0.5 × a × b2, where a and b represent the long and short diameters of the tumor, respectively. On the 21st day, mice were euthanized. The dissected tumor samples from PDX models were then subjected to the following real-time PCR assays and CUT&Tag-seq assays. The informed consent from all participants were acquired. The use of female mice in the PDX assay is due to the fact that the PDX donor for this experiment was female. Female mice were chosen to maintain experimental consistency and to avoid potential sex-specific factors that could confound the results. This experimental protocol was approved by the Institutional Animal Care and Use Committee (IACUC) of Shanghai GeneChem Organism and the Ethics Committee of Renji Hospital, Shanghai Jiao Tong University School of Medicine.

Wild animals	None
Reporting on sex	The findings apply to both sexes. Female mice were used in the PDX assay because the PDX donor for this experiment was female. Conversely, male mice were used in the CDX assays with a cancer cell line derived from a male donor. Maintaining consistency of sex between the host and xenograft ensures experimental consistency and helps avoid potential confounding factors.
Field-collected samples	None
Ethics oversight	All animal experiments were conducted in accordance with the guidelines approved by Institutional Animal Care and Shanghai Jiao Tong University Animal Care commission.

Note that full information on the approval of the study protocol must also be provided in the manuscript.
